# Association of *LPCAT1-*rs8352 genetic variant with susceptibility and severity of pediatric bronchial asthma: a case-control study

**DOI:** 10.1186/s12887-025-05425-x

**Published:** 2025-01-27

**Authors:** Khalid M. Mohany, Yasser Gamal, Yaser F. Abdel Raheem

**Affiliations:** 1 Department of Medical Biochemistry and Molecular Biology, Faculty of Medicine, Assiut University, EL Gammaa street, Assiut, 71515 Egypt; 2 Department of Pediatrics, Faculty of Medicine, Assiut University, Egypt, 71515 Assiut Egypt

**Keywords:** *LPCA1*-rs8352, Genotypes, Pediatric Asthma, Asthma severity

## Abstract

**Background:**

This study aimed to investigate the possible association of *LPCAT1-*rs8352 genetic variant (single nucleotide change C to G) with the onset and severity of pediatric asthma. Additionally, the study examined the influence of *LPCAT1-*rs8352 genotypes on asthma-related biomarkers including blood eosinophils count (BEC), eosinophil cationic protein (ECP), high-sensitivity C-reactive protein (hs-CRP), and immunoglobulin E (IgE) and on lung function [forced expiratory volume in one second (FEV1) and the forced vital capacity (FVC)].

**Patients and methods:**

The study included ninety-six participant grouped into two groups: G1 (46 asthmatics) and G2 (50 healthy controls). ECP, hs-CRP, and total IgE serum levels were measured using their corresponding ELISA kits. Neonatal blood DNA was extracted using the Gene JET™ Whole Blood Genomic DNA Purification Mini Kit. Genotyping was performed by RT-PCR.

**Results:**

A significantly higher proportion of individuals in G1 had the LPCAT1-rs8352 CC and GC genotypes compared to G2 (*p* < 0.001). Individuals with the CC genotype exhibited significantly more severe asthma, along with elevated levels of BEC, ECP, hs-CRP, and total IgE. Those with the GC genotype demonstrated a similar, though less severe, pattern, followed by individuals with the GG genotype. The FEV1 and FVC values showed the opposite trend, with individuals having the GG genotype exhibiting the highest lung function values.

**Conclusion:**

The *LPCAT1*-rs8352 allele C is associated with pediatric asthma onset and severity. Further research on the *LPCAT1* genetic variants may provide a deeper understanding of pediatric bronchial asthma mechanisms and lead to improved management strategies.

## Background

Bronchial asthma (BA) imposes a substantial burden on the healthcare system. It is the most common chronic respiratory disease and a leading cause of pediatric illness, with up to half of all asthmatics experiencing their initial symptoms during childhood [1, 2]. BA is associated with persistent airway inflammation, bronchial hyperactivity with bronchoconstriction, cough, wheezing, dyspnea, and chest tightness with a declined pulmonary function (e.g., decreased forced expiratory volume at 1 s [FEV1]) and response to pharmacotherapy.

While the exact cause of pediatric asthma remains unknown, it is likely a combination of genetic and environmental factors. Individuals with a family history of asthma or allergies, or those exposed to secondhand smoke or air pollution, are at a higher risk [3–6]. Although the precise influence of genetics on asthma remains unclear, genetic variations may explain why asthma rates vary across the globe among different ethnic groups [7]. Additionally, single nucleotide polymorphisms (SNPs) have been associated with the timing of asthma onset, reduced lung function, and the infiltration of the airways by immune cells such as CD4 + cells and eosinophils [7–9]. Genetic differences may partially account for why men and women experience asthma differently [10].

Lipid metabolism plays a crucial role in the development of asthma [11, 12]. Lysophosphatidylcholine acyltransferase 1 (*LPCAT1*) and its protein product have been extensively investigated for their potential role in various lung diseases, including emphysema, fibrosis, pneumonia, and neonatal respiratory distress syndrome [13–16]. LPCAT enzymes play a crucial role in phospholipid metabolism and lung surfactant production [17, 18]. Found in various tissues, LPCATs catalyze the transfer of an acyl group to lysophosphatidylcholine (LPC), converting it back to phosphatidylcholine (PC) [17]. Three primary SNPs have been identified within the *LPCAT1* gene: *LPCAT1*-rs8352, *LPCAT1*-rs9728, and *LPCAT1*-rs10630 [16].

To our knowledge, no research has examined the potential link between these variations, their products, and the susceptibility, onset, or severity of bronchial asthma either in children or in adults. Therefore, this study aimed to investigate the possible association of one of these SNPs (*LPCAT1-*rs8352) with the onset and severity of childhood asthma. Additionally, the study aimed to explore the influence of *LPCAT1-*rs8352 genotypes on asthma-related biomarkers, including blood eosinophils count (BEC), eosinophil cationic protein (ECP), high-sensitivity C-reactive protein (hs-CRP), and immunoglobulin E (IgE), and on lung function as measured by FEV1 and forced vital capacity (FVC).

## Materials and methods

### Participants recruitment

This case-control study was conducted at Assiut University Pediatrics Hospital, recruiting all patients diagnosed with bronchial asthma who presented consecutively at the facility alongside healthy control volunteers. The study was reviewed and authorized by the local ethics board at the Faculty of Medicine, Assiut University (IRB#17300776). Written informed consent was obtained from the children’s guardians.

Ninety-six participants aged 7 to 13 years (28 male and 68 female) participated in the study. They were divided into two groups: Group 1 (G1, cases, 46 participant diagnosed with asthma) and Group 2 (G2, controls; 50 healthy participant). Both groups were age- and sex-matched. G1 was followed in the hospital’s Pediatric Allergy and Asthma Unit, while controls had no history of asthma and were seen for other reasons in the outpatient department.

A complete medical history was taken from all individuals, with particular emphasis on family history of bronchial asthma, chronic exposure to dust, smoking, or other potential allergens. For the G1 participants, we additionally inquired about the number of days and/or nights they experienced asthma symptoms. This information, along with spirometry results, was used to classify their asthma severity according to established criteria [19]. Additionally, all participants underwent careful clinical examinations and investigations to rule out any other conditions. Body mass index (BMI) was calculated for all participants using the equation $\:\frac{Weight\:\left[Kg\right]}{Height\:\left[m2\right]}$. Chest X-ray was helpful in differentiating asthma from other conditions mimicking its symptoms. In addition, all participants underwent pulmonary function testing using spirometry.

The International Study of Asthma and Allergies in Childhood (ISAAC) questionnaire was used to assess asthma symptoms (wheeze, cough, shortness of breath) and risk factors [20]. Participants who experienced these symptoms, demonstrated a positive response to bronchodilators (increased FEV1, reduced wheezing and coughing, and Improved breathing), or gave a history of a positive response to inhaled or oral corticosteroids were diagnosed with asthma. Asthma severity was assessed based on Global Initiative for Asthma (GINA) guidelines and control status was evaluated [21]. Lung function tests (spirometry) were performed according to the American Thoracic Society (ATS) standards to confirm airflow limitation with bronchodilator trials [22, 23].

A Spirobank II device (MIR Italy, SN: A23-0Y) was used for the lung function tests. We measured FEV1 and FVC and adjusted the results for weight, height, and body mass index (percentage predicted). Healthy participants demonstrated normal lung function, with FEV1 values exceeding 80% predicted and FEV1/FVC ratios within the normal range. They showed minimal to no response to bronchodilator medication and reported no breathing difficulties [24]. Participants experiencing asthma exacerbation requiring steroids in the past 3 months or with other systemic or pulmonary diseases were excluded [25].

Based on the frequency and severity of symptoms, asthma is classified into four categories: intermittent, mild persistent, moderate persistent, and severe persistent. Intermittent asthma, the least severe form, occurs less than two days per week. Patients may experience nighttime symptoms less than twice a month, have a normal FEV1 (85% or more), and require a rescue inhaler less than twice a week. Mild persistent asthma is characterized by daytime symptoms more than twice a week but less than daily, nighttime symptoms three to four times a month, and a slightly reduced FEV1 (80% or more). Minor limitations on daily activities and a need for rescue inhaler more than twice a week but less than daily are common. Moderate persistent asthma involves more frequent daytime symptoms, nighttime symptoms more than once a week, a reduced FEV1 (60–80% without treatment), and some limitations on daily activities. Rescue inhaler use is required daily. Severe persistent asthma, the most severe form, is characterized by persistent symptoms, frequent nighttime symptoms, a significantly reduced FEV1 (less than 65%), and severe limitations on daily activities. Rescue inhaler use is required multiple times per day [21–23, 26, 27].

### Sampling and laboratory analysis

### Approximately ten ml of venous blood was drawn from each participant

**Sera collection**: Four milliliters of the blood were collected in a plain test tube and allowed to clot at room temperature for 15 min. This sample was then centrifuged for 15 min at 3,000 rpm. The resulting serum was separated and stored in aliquots at -20 °C until analysis. Serum levels of glucose, liver enzymes, kidney function markers, and glycated hemoglobin were analyzed using standard laboratory methods in the outpatient clinic’s lab. Additionally, enzyme-linked immunosorbent assays (ELISAs) were performed to measure serum levels ECP, hs-CRP, and total IgE. The specific ELISA kits used were obtained from MyBioSource, Inc. (San Diego, CA, USA): Cat# MBS2602477 for ECP, Cat# MBS703598 for hs-CRP, and Cat# MBS564049 for IgE.

#### Whole blood collection

the remaining six ml of blood were used for a complete blood count, glycated hemoglobin analysis, and genetic work.

### Genotyping

Genomic DNA was extracted from venous blood using the GeneJET™ Whole Blood Genomic DNA Purification Mini Kit (Thermo Fisher Scientific, USA, Cat# 0781). The *LPCAT1-*rs8352 SNP genotyping was performed using quantitative real-time PCR (RT-PCR) on an Applied Biosystems 7500 Fast Real-Time PCR System (version 2.3 software). This assay required purified genomic DNA, a pre-designed TaqMan^®^ SNP Genotyping Assay specific for rs8352, and TaqMan^®^ Genotyping Master Mix. The reactions were run on 96-well plates in 25 µL volumes containing TaqMan™ Universal Master Mix II (no UNG), TaqMan^®^ Pre-Designed SNP Genotyping Assay (20X), purified DNA sample, and TE buffer (1X). All analyses were automated with default settings. The PCR cycling conditions involved an initial activation step at 95 °C for 10 min, followed by 45 cycles of denaturation at 95 °C for 15 s and annealing/extension at 60 °C for 1 min. The TaqMan^®^ SNP Genotyping Assay utilizes sequence-specific primers and two fluorescently labeled probes: a VIC^®^-dye labeled probe for Allele 1 and a FAM™-dye labeled probe for Allele 2.

The TaqMan™ Universal Master Mix II (no UNG) contains AmpliTaq Gold^®^ DNA polymerase, ultrapure dNTPs, ROX™ passive reference dye, and optimized buffer components. We used a pre-designed TaqMan^®^ SNP Genotyping Assay specific for rs8352, located on chromosome 5 at position 146,293,638 (GRCh38 build). The assay targets the following context sequence (VIC/FAM): ACCTCCCCAGGGGACCCAGCGGAAG[C/G]AGAGGGATTACCTGCTGGAGAGAGG, where the variant is a C/G transversion substitution.

### Statistical analysis

Data analysis was performed using SPSS software (version 26). Normality of continuous variables was assessed with the Shapiro-Wilk test. For normally distributed data, Student’s t-tests compared data between two groups, while one-way ANOVA was used for comparisons among three or more groups. Categorical data were compared using Chi-square tests [28]. A p-value ≤ 0.05 was considered statistically significant.

## Results

A higher percentage of participants with a positive family history of asthma and exposure to dust was observed in BA group (G1) than in the control group (G2) (Table 1). Also, the BA group (G1) also showed a significantly higher percentage of the *LPCAT1*-rs8352 GC and CC genotypes compared to G2 (Table 1).

**Table 1:** Key medical features and *LPCAT1-*rs8352 genotypes of the studied groups

		G1 Bronchial asthma group [*n* = 46]	G2 Healthy control group [*n* = 50]	*p*-Value
**Gender**	**Male**	13 [28.3%]	15 [30%]	0.515
	**Female**	33 [71.7%]	35 [70%]	
Age [years]*		9.9 ± 1.6	10.6 ± 1.7	0.06
**BMI [kg/m** ^**2**^ ]*		19.8 ± 0.7	19.9 ± 0.8	0.569
**Family history of bronchial asthma**	**Yes**	19 [41.3%]	5 [10%]	< 0.001
	**No**	27 [58.7%]	45 [90%]	
**Chronic exposure to aeroallergens**	**Yes**	27 [58.7%]	12 [24%]	0.001
	**No**	19 [41.3%	38 [76%]	
***LPCAT1-*** **rs8352 genotypes**	**GG**	4 [8.7%]	26 [52%]	< 0.001
	**GC**	23 [50%]	16 [32%]	
	**CC**	19 [41.3%]	8 [16%]	

*Data were presented as mean ± SD and compared by Student t test, the other data were presented as n [%] compared by Chi-square test, **BMI**: Body mass index

Moreover, BA group (G1) also showed significantly higher levels of ECP, hs-CRP, and total IgE and lower FEV1, FVC (% predicted) and FEV1/FVC ratio compared to control group (G2) (Table 2).

Importantly, no significant differences were found between the two groups in terms of age, gender, BMI, glycemic indices, liver function tests, or kidney function tests (Tables 1 and 2), confirming successful group matching for baseline characteristics.

**Table 2:** Laboratory analyses and spirometry results of the study groups

		G1 Bronchial asthma group [*n* = 46]	G2 Healthy control group [*n* = 50]	*p*-Value
**RBG (mg/dl)**		101.2 ± 13.0	104.4 ± 12.1	0.124
**HBA1c%**		5.1 ± 0.4	5.2 ± 0.3	0.356
**BUN (mg/dl)**		26.84 ± 7.64	26.64 ± 8.84	0.621
**sCr (mg/dl)**		0.65 ± 0.22	0.67 ± 0.25	0.528
**sALT (U/l)**		12.8 ± 4.1	12.5 ± 3.4	0.683
**sAST (U/l)**		14.9 ± 3.8	14.7 ± 4.0	0.604
**WBCs count (10** ^**3**^ **/ml)**		7.5 ± 2.1	6.3 ± 1.5	< 0.001
**Neutrophils count (10** ^**3**^ **/ml)**		3.2 ± 0.9	3.2 ± 1.4	0.847
**BEC/ml**		387.7.7 ± 147.3	152.4 ± 64.1	< 0.001
**ECP [µg/ml]**		28.6 ± 11.3	18.9 ± 12.6	< 0.001
**hs-CRP [mg/L]**		1.1 ± 0.6	0.58 ± 0.2	< 0.001
**Total IgE [IU/ml]**		501.9 ± 105.1	120.1 ± 48.2	< 0.001
**FEV1** **(%predicted)**	**Before bronchodilators**	73.6 ± 9.1	99.2 ± 5.2	< 0.001
	**After bronchodilators**	87.1 ± 6.5	95.6 ± 7.9	< 0.001
**FVC** **(%predicted)**	**Before bronchodilators**	86.8 ± 4.3	97.9 ± 8.3	< 0.001
	**After bronchodilators**	93.9 ± 4.6	98.3 ± 7.9	< 0.001
**FEV1/FVC** **ratio**	**Before bronchodilators**	80.5 ± 10.7	91.7 ± 9.8	< 0.001
	**After bronchodilators**	86.4 ± 12.9	92.2 ± 11.1	0.004

**RBG**: Random blood glucose, **HBA1c**: Glycated hemoglobin, **BUN**: Blood urea nitrogen, **sCr**: Serum creatinine, **sALT**: Serum alanine transaminase, **sAST**: Serum aspartate transaminase, **WBCs**: White blood cells, **BEC**: Blood eosinophils count, **ECP**: Serum eosinophil cationic protein, **hs-CRP**: Serum high-sensitivity C-reactive Protein, **IgE**: Immunoglobulin E. **FEV**_**1**_: Forced expiratory volume in the first second, **FVC**: forced expiratory volume

Within the BA group (G1), 16 (16.7%) had mild intermittent asthma, 13 (13.5%) had mild persistent asthma, 13 (13.5%) had moderate persistent asthma, and 4 (4.3%) had severe persistent asthma. Patients with severe persistent asthma exhibited significantly lower FEV1 and FVC (% predicted) and higher ECP, hs-CRP, and total IgE levels compared to those in the other asthma severity groups (Table 3).

**Table 3:** Pediatrics’ characteristics, laboratory analyses, and spirometry results according to the degree of asthma severity

	Degree of asthma severity				*p*-Value
	Mild intermittent [*n* = 16]	Mild persistent [*n* = 13]	Moderate persistent [*n* = 13]	Severe persistent [*n* = 4]	
**FEV**_**1**_ **[% predicted]**	90.8 ± 8.2	85.5 ± 4.7	73.9 ± 3.9	60.9 ± 2.4	< 0.001
**FVC (%predicted)**	91.7 ± 6.3	84.2 ± 3.5	75.4 ± 3.3	63.5 ± 1.8	< 0.001
**BEC /ml**	236 ± 74.2	372 ± 60.4	560 ± 63.8	611 ± 4.8	< 0.001
**ECP [µg/ml]**	19.8 ± 6.2	26.1 ± 1.4	33.9 ± 6.9	55 ± 4.7	< 0.001
**hs-CRP [mg/L]**	0.7 ± 0.2	1 ± 0.1	1.4 ± 0.4	2.5 ± 0.3	< 0.001
**Total IgE [IU/ml]**	391.6 ± 62.3	502.9 ± 47.8	587.7 ± 39.7	661 ± 10.6	< 0.001

**FEV**_**1**_: Forced expiratory volume in the first second, **FVC**: forced vital capacity, **BEC**: blood eosinophils count, **ECP**: Serum eosinophil cationic protein, **hs-CRP**: Serum high-sensitivity C-reactive protein, **IgE**: Immunoglobulin E

A significantly higher percentage of individuals with a family history of bronchial asthma were found to be associated with the *LPCAT1-*rs8352 allele C (CC > GC > GG). Similar results were observed for asthma severity, and levels of BEC, ECP, hc-CRP, and total IgE (higher levels were associated with allele C). Conversely, significantly lower lung function values (FEV1 and FVC) were associated with allele C (Table 4).

**Table 4:** Pediatrics’ characteristics, laboratory analyses, and spirometry results according to the *LPCAT1-*rs8352 genotypes

		LPCAT1-rs8352 GG genotype [*n* = 4]	LPCAT1-rs8352 GC genotype [*n* = 23]	LPCAT1-rs8352 CC genotype [*n* = 19]	*p*-Value
Gender*	**Male**	0 [0.0%]	6 [26.1%]	7 [36.8%]	0.314
	**Female**	4 [100%]	17 [73.9%]	12 [63.2%]	
Family history of bronchial asthma*	**Yes**	2 [50%]	8 [34.7%]	9 [47.4%]	0.010
	**No**	2 [50%]	15 [65.3%]	10 [52.6%]	
**Child’s age [years]**		9.4 ± 2.0	10.2 ± 1.9	9.7 ± 1.5	0.533
**BMI [kg/m** ^**2**^ **]**		18.6 ± 0.7	18.9 ± 0.8	18.7 ± 0.6	0.631
Degree of asthma severity*	**Mild intermittent**	4 [100.0%]	9 [39.1%]	3 [15.8%]	0.003
	**Mild persistent**	0 [00.0%]	10 [43.5%]	3 [15.8%]	
	**Moderate persistent**	0 [00.0%]	3 [13.1%]	10 [52.6%]	
	**Severe persistent**	0 [00.0%]	1 [4.3%]	3 [15.8%]	
**FEV**_**1**_ **[% predicted]**		93.8 ± 5.2	78.5 ± 3.3	61.8 ± 2.4	< 0.001
**FVC (%predicted)**		92.7 ± 6.3	73.2 ± 3.5	60.5 ± 1.8	< 0.001
**BEC /ml**		184.9 ± 89.4	351.7 ± 128.9	534.2 ± 185.1	< 0.001
**ECP [µg/ml]**		13.8 ± 9.8	27.4 ± 13.7	42.2 ± 24.5	0.008
**hs-CRP [mg/L]**		0.6 ± 0.2	0.9 ± 0.4	1.4 ± 0.6	0.001
**Total IgE [IU/ml]**		340.6 ± 83.1	503.9 ± 104.1	647.9 ± 126.7	< 0.001

*Data were presented as n [%] and compared by Chi-square test; the other data were presented as mean ± SD and compared by one way ANOVA. **BMI**: body mass index, **BEC**: blood eosinophils count, **ECP**: Serum eosinophil cationic protein, **hs-CRP**: Serum high-sensitivity C-reactive protein, **IgE**: Immunoglobulin E

Within the BA group, comparing individuals with the LPCAT1-rs8352 GG genotype to those with the GC + CC genotypes, the latter showed significantly more severe asthma (*p* = 0.042). Additionally, the levels of BEC, ECP, hs-CRP, and serum total IgE were significantly higher in the GC + CC group compared to the GG-genotype group (p values = 0.009, 0.014, < 0.001, and 0.005, respectively). Conversely, lung functions (FEV1 and FVC) were significantly reduced in the GC + CC group compared to individuals with the GG genotype (*p* < 0.001 for both).

When comparing individuals with the LPCAT1-rs8352 GG + GC genotypes to those with the CC genotype within the BA group, the latter again demonstrated significantly more severe asthma (*p* = 0.003). Furthermore, levels of BEC, ECP, hs-CRP, and serum total IgE were significantly higher in the CC group compared to the GG + GC group (p values = < 0.001, 0.011, 0.002, and < 0.001, respectively). In contrast, lung functions (FEV1 and FVC) were significantly reduced in the CC group compared to individuals with the GG + GC group (*p* < 0.001 for both).

## Discussion

Genetic factors significantly influence both the onset and progression of asthma [3, 4, 29]. Certain genetic variants may increase the risk of developing asthma to a greater degree than others [30]. Our findings showed that individuals with allele C at the *LPCAT1-*rs8352 gene (i.e., GC and CC genotypes) had a higher risk of developing bronchial asthma compared to those with the GG genotype. Furthermore, the presence of the allele C was associated with both the severity of asthma and the percentage of children with a family history of bronchial asthma.

The *LPCAT1* gene encodes the first isozyme in the LPCAT protein family, found in various tissues like the lungs, liver, and fat. LPCAT is the primary enzyme involved in cell membranes biogenesis and a crucial enzyme in the production of surfactant. LPCAT1 is situated on chromosome 5p15.33 and contains 18 exons. The rs8352 SNPs are located in the 3’-untranslated region (UTR) of exon 14 of the *LPCAT1* gene. These SNPs could be functionally significant due to the known role of the 3’-UTR in regulating mRNA stability and how efficiently it is translated into a protein [16].

Interestingly, LPCATs also play a critical role in signaling pathways that regulate cellular inflammatory responses by affecting membrane phospholipids [31]. Disruptions in LPCAT-related pathways have been implicated in the development of various diseases, including respiratory distress syndrome, diabetes mellitus, high cholesterol, non-alcoholic fatty liver disease, inflammatory disorders, and even some cancers [32–35].

Furthermore, LPC itself contributes to inflammation in different tissues. It increases the expression of molecules like intercellular adhesion molecule-1 (ICAM-1) and vascular cell adhesion molecule-1 (VCAM-1) on cell surfaces. These molecules help monocytes stick to the lining of blood vessels, facilitating their migration into inflamed tissues. Additionally, LPC can induce the production of interferon by T-lymphocytes [36]. Also, the LPC may harm the pulmonary tissues through the increase in the capillary permeability, altering surfactant features, induction of airway mast cell secretions, and hyper-responsiveness [36, 37]. Also, it desensitizes the β-adrenergic receptor in the bronchial smooth muscles and enhances bronchial infiltrations by eosinophils and triggers the bronchial constrictions [38]. The LPC concentration in the bronchoalveolar lavage fluid increased significantly in cases with atopic asthma [37].

The current study also revealed that individuals with the *LPCAT1-*rs8352 CC genotype exhibited higher blood levels of BEC, ECP, hs-CRP, and total IgE, compared to individuals with the GC and GG genotypes. Additionally, individuals with the GC genotype had significantly higher levels of these markers compared to those with the GG genotype.

Eosinophils are primary cells driving tissue damage in bronchial asthma. They are believed to contribute to bronchitis and desquamation of the bronchial mucosa in asthmatic patients. Eosinophils release various proteins that might be involved in these processes, including the ECP, eosinophil peroxidase (EPO), and major basic protein (MBP). In vitro studies (laboratory experiments) have shown that ECP is toxic to the bronchial epithelium, the lining of the airways. This suggests that ECP may increase the damaging effects of eosinophils on the bronchial lining in asthma [39, 40].

A study by Rydell et al. found significantly higher blood levels of BEC and ECP in individuals with bronchial asthma compared to healthy individuals. Interestingly, the study also showed that ECP levels were effective in distinguishing individuals with current asthma from healthy controls. This was demonstrated using receiver operating characteristic curve (ROC) curve analysis, which yielded an area under the curve (AUC) of 80%. The analysis also determined a cutoff point of 29 µg/ml for ECP levels, with a sensitivity of 70.3% and a specificity of 81.4% [41].

Elevated levels of hs-CRP, a marker of systemic inflammation, are found in asthmatics, even those with mild symptoms, suggesting a potential role for low-grade inflammation in asthma severity [42]. Likewise, IgE, an antibody involved in allergic reactions, is elevated in allergic asthma, reflecting its contribution to asthma attacks [43]. Previous studies have confirmed these findings, demonstrating higher hs-CRP and IgE in asthmatics compared to healthy controls, particularly in allergic asthma. These markers are associated with asthma severity and have potential as tools for predicting asthma control. Together, hs-CRP and IgE offer insights into the complex interplay between inflammation and allergic responses in asthma [42, 44].

## Conclusion

Our findings suggest an association between the allele C of the *LPCA1-*rs8352 gene variant and both the development and severity of pediatric asthma. Individuals carrying the CC genotype at the *LPCA1-*rs8352 variant exhibited significantly worse asthma severity. This was evidenced by higher levels of blood markers like BEC, ECP, hs-CRP, and total IgE, alongside lower lung function measured by FEV1 and FVC values than individuals with the GC and GG genotypes.

### Limitation of the study

The main limitation of this study is the relatively small sample size. Additionally, our analysis didn’t focus on the broader genetic makeup of the population. To address these limitations, we recommend a nationwide study to investigate the distribution of *LPCA1-*rs8352 and other bronchial asthma’s relevant SNPs within the population. This larger study could then explore potential associations between these genetic variations and both the onset and severity of asthma.

## Data Availability

The datasets analyzed during the current study are available in the European Variation Archive repository, Assembly accession GCA_000001405.27 (GRCh38.p12), Chromosome/Contig accession CM000667.2, The European Bioinformatics Institute < EMBL-EBI.

## Abbreviations

LPCAT1: Lysophosphatidylcholine acyltransferase 1 gene
FEV1: Forced expiratory volume in the first second
SNPs: Single Nucleotide Polymorphisms
CD4+: Cluster of differentiation 4
LPC: Lysophosphatidylcholine
PC: Phosphatidylcholine
BEC: Blood eosinophils count
ECP: Eosinophil cationic protein
hs-CRP: High-sensitivity C-reactive protein
IgE: Immunoglobulin E
IRB: Institutional Review Board
BMI: Body mass index
GINA: Global Initiative for Asthma
ISAAC: International Study of Asthma and Allergies in Childhood
FVC: Forced vital capacity
ELISA: Enzyme-linked immunosorbent assay
RT-PCR: Real-time polymerase chain reaction
ANOVA: Analysis of variance
ROC: The receiver operating characteristic curve
AUC: Area under the receiver operating characteristic curve
MBP: Major basic protein
EPO: Eosinophil peroxidase
ICAM: Intercellular adhesion molecule
VECAM: Vascular cell adhesion molecule
